# The effect of protein acetylation on the formation and processing of inclusion bodies and endogenous protein aggregates in *Escherichia coli* cells

**DOI:** 10.1186/s12934-016-0590-8

**Published:** 2016-11-10

**Authors:** Dorota Kuczyńska-Wiśnik, María Moruno-Algara, Karolina Stojowska-Swędrzyńska, Ewa Laskowska

**Affiliations:** Department of General and Medical Biochemistry, Faculty of Biology, University of Gdansk, Wita Stwosza 59, 80-308 Gdansk, Poland

**Keywords:** Lysine acetylation, Recombinant proteins, Inclusion bodies, Protein aggregates

## Abstract

**Background:**

Acetylation of lysine residues is a reversible post-translational modification conserved from bacteria to humans. Several recent studies have revealed hundreds of lysine-acetylated proteins in various bacteria; however, the physiological role of these modifications remains largely unknown. Since lysine acetylation changes the size and charge of proteins and thereby may affect their conformation, we assumed that lysine acetylation can stimulate aggregation of proteins, especially for overproduced recombinant proteins that form inclusion bodies.

**Results:**

To verify this assumption, we used *Escherichia coli* strains that overproduce aggregation-prone VP1GFP protein. We found that in Δ*ackA*-*pta* cells, which display diminished protein acetylation, inclusion bodies were formed with a delay and processed faster than in the wild-type cells. Moreover, in Δ*ackA*-*pta* cells, inclusion bodies exhibited significantly increased specific GFP fluorescence. In CobB deacetylase-deficient cells, in which protein acetylation was enhanced, the formation of inclusion bodies was increased and their processing was significantly inhibited. Similar results were obtained with regard to endogenous protein aggregates formed during the late stationary phase in Δ*ackA*-*pta* and Δ*cobB* cells.

**Conclusions:**

Our studies revealed that protein acetylation affected the aggregation of endogenous *E. coli* proteins and the yield, solubility, and biological activity of a model recombinant protein. In general, decreased lysine acetylation inhibited the formation of protein aggregates, whereas increased lysine acetylation stabilized protein aggregates. These findings should be considered during the designing of efficient strategies for the production of recombinant proteins in *E. coli* cells.

**Electronic supplementary material:**

The online version of this article (doi:10.1186/s12934-016-0590-8) contains supplementary material, which is available to authorized users.

## Background

*Escherichia coli* is by far the most widely used host for the production of recombinant proteins. There are many protocols, a great number of expression plasmids, and engineered strains that can be used to obtain high protein yields [1]. Despite numerous advantages, biotechnological potential of *E. coli* also has its limitations including the risk of protein aggregation and accumulation of acetate as by-product. Upon high-level production, recombinant proteins may aggregate and form inclusion bodies (IBs) in *E. coli* cells. Formation of IBs is a complex and dynamic process affected by various factors involving molecular chaperones that control protein folding. The vast increase in misfolded recombinant proteins exhausts the capacity of molecular chaperones leading to the formation of IBs [2, 3]. To recover biologically active proteins from IBs, additional steps in purification procedure such as solubilization of IBs and subsequent refolding of desired proteins are required. Coexpression of molecular chaperones or lower production rate can prevent aggregation and facilitate the proper folding of recombinant proteins [1–3]. A whole spectrum of polypeptides with different conformations, including partially or even fully native structures, can be found in IBs [4–6]. Therefore, in some cases, insoluble but active proteins sequestered in IBs can be the preferred form of the product [7].

Another obstacle in recombinant protein production in *E. coli* cells is accumulation of acetate during extensive aerobic fermentation in cultures supplemented with glucose. At higher glucose concentrations, the carbon flux into the cells exceeds tricarboxylic acid cycle capacity, and acetyl-CoA is converted into acetate, which is easily excreted from the cell. The excretion of acetate into the environment results also from the need to regenerate the NAD^+^ consumed by glycolysis and to recycle the CoA that is required to convert pyruvate to acetyl-CoA [8]. A high concentration of acetate, externally added or excreted from the cell, limits the growth of *E. coli*. Undissociated or acidic forms of acetate permeate the membranes and dissipate the transmembrane pH gradient. After acetate dissociation, the proton acidifies the cytoplasm, while the anion increases the internal osmotic pressure. Acetate toxicity results also from the depletion of the intracellular methionine pool, with the concomitant accumulation of the toxic intermediate homocysteine [9]. Various strategies have been developed to limit acetate accumulation or reduce its negative effects [10]. One of such strategies is reduction of acetate concentration and improvement of protein production achieved by deletion or downregulation of the *ackA*-*pta* pathway using antisense-RNA strategy [11, 12]. The *ackA*-*pta* operon encodes phosphate acetyltransferase (Pta), which converts acetyl-CoA and inorganic phosphate to acetyl-phosphate (AcP), and acetate phosphotransferase (AckA), which converts acetyl phosphate to acetate and ATP. Downregulation or deletion of the *ackA*-*pta* reduces both acetate level and also the concentration of AcP. Interestingly, it has been demonstrated that the Δ*ackA*-*pta* strain was defective in the degradation of model unstable proteins and accumulated increased levels of protein aggregates formed upon heat stress [13]. Furthermore, it was found that the Δ*ackA*-*pta* mutation resulted in reduced refolding and disaggregation of heat-denatured luciferase [14]. The authors proposed that the presence of AcP is required for efficient proteolysis and function of molecular chaperones in *E. coli* cells. The exact role of AcP in the protein quality-control system remains, however, unknown. Recent studies have demonstrated that AcP participates in non-enzymatic N^ε^-lysine acetylation in *E. coli* [15]. Acetylation of lysine residues is a conserved post-translational reversible modification that occurs in all three kingdoms. In eukaryotes protein lysine acetylation regulates diverse physiological processes such as cell cycle, cell morphology, protein synthesis, mRNA splicing, and central metabolism. Contrary to eukaryotes, the impact of protein acetylation on bacterial physiology is poorly understood; however, global proteomic studies revealed acetylation of thousands of lysine residues in hundreds of *E. coli* proteins involved in various cellular processes [16]. Apart from non-enzymatic acetylation by AcP, *E. coli* proteins can be modified by lysine acetyltransferase PatZ (the *pka* gene product), which transfers acetyl group from acetyl-CoA to N^ε^ lysine residues. N^ε^-lysine acetylation can be reversed by the deacetylase CobB regardless of acetylation mechanism [17].

N^ε^-lysine protein acetylation is stimulated in media supplemented with glucose or acetate [16, 18]. We demonstrated previously that acetate or glucose enhanced protein aggregation in stationary *E. coli* cultures [19]. We assume that increased protein aggregation in cells exposed to a high concentration of glucose or acetate can result not only from the aforementioned toxic effects of acetate but also from *N*-lysine acetylation. Several lines of evidence exist to support this notion. Neutralization of the charge on lysine residues by acetylation has been shown to induce aggregation of modified lysozyme in vitro [20]. Whereas acetylation of the microtubule-associated protein tau inhibited tau function via impaired tau–microtubule interactions and promotes pathological tau aggregation in neurons [21].

Altogether, these data suggest that the formation of inclusion bodies and biological activities of proteins sequestered in the aggregates can be affected by acetylation. This is an important issue to address because recombinant proteins are often produced under acetylation- or deacetylation-favorable conditions (e.g., glucose-supplemented media or acetate-deficient engineered strains); however, the effect of acetylation on recombinant products has not been investigated yet. To verify our hypothesis, we analyzed the formation and processing of IBs and aggregates of endogenous proteins in two bacterial strains: Δ*ackA*-*pta* cells, which are characterized by diminished protein acetylation, and in CobB-deficient cells (Δ*cobB*) with enhanced acetylation.

## Results

### Overproduction of VP1GFP and formation of IBs are enhanced in Δ*cobB* cells and inhibited in the Δ*ackA*-*pta* strain

**Fig. 1:**
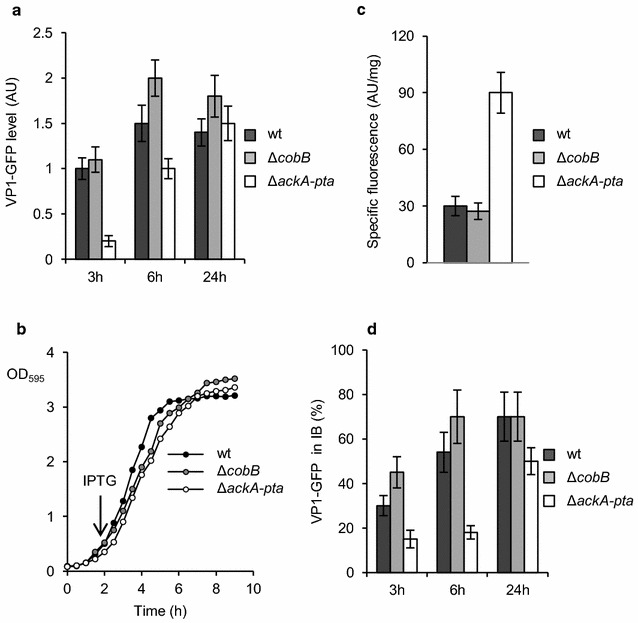
Overproduction of VP1GFP in mutant strains defective in deacetylation (Δ*cobB*) or acetylation (Δ*ackA*-*pta*). Bacteria were grown at 37 °C in LB medium supplemented with 100 µg/ml ampicillin. 1 mM IPTG was added at an OD_595_ = 0.5 to induce the synthesis of VP1GFP. **a** After 3, 6, and 24 h, whole-cell extracts were resolved by SDS-PAGE, subjected to immunodetection using anti-GFP antibodies and analyzed by densitometry to estimate the relative level of VP1GFP. **b** Representative growth curves of wt, Δ*cobB,* and Δ*ackA*-*pta* cells. **c** GFP fluorescence in whole-cell extracts was recorded after 3 h of induction using EnSpire plate reader (PerkinElmer). Specific fluorescence emission of VP1GFP was estimated as described in the “Methods” section. **d** The amounts of VP1GFP in IBs were calculated in relation to the total VP1GFP level in bacterial extracts (100%). IBs were isolated from *E. coli* cells after induction of VP1GFP synthesis by 1 mM IPTG at the times indicated in the figure. Error bars represent the standard deviation of three values. AU-arbitrary units

First, we analyzed the formation and processing of IBs containing the fusion VP1GFP protein, which consists of GFP and foot-and-mouth disease virus VP1 capsid protein, and retains its fluorescence in IBs [22]. We found that the overproduction of VP1GFP was enhanced in the Δ*cobB* strain and inhibited in Δ*ackA*-*pta* cells compared to wild-type (wt) cells (Fig. 1a). VP1GFP synthesis inhibited the growth of wt and Δ*cobB* but not Δ*ackA*-*pta* cells. Just before isopropyl β-d-1-thiogalactopyranoside (IPTG) induction, Δ*ackA*-*pta* cells were found to grow slower than wt strain (Fig. 1b). After IPTG induction, the generation time increased from 33 to 44 min in the wt culture and, surprisingly, decreased from 53 to 44 min in the Δ*ackA*-*pta* culture. Overproduction of VP1GFP affected the growth of Δ*cobB* cells—its generation time increased after IPTG induction from 33 to 54 min. It should be noted, however, that the Δ*cobB* culture reached the stationary phase later than wt cells (Fig. 1b). Interestingly, specific fluorescence of VP1GFP in the Δ*ackA*-*pta* strain was threefold higher than in wt and Δ*cobB* cells (Fig. 1c). Presumably, slower VP1GFP synthesis in Δ*ackA*-*pta* cells gives the recombinant protein time to fold properly and resulted in higher fluorescence. In accordance with these data, we found that in cells with impaired AckA-Pta pathway, the formation of IBs was postponed. On the other hand, faster accumulation of IBs was observed in Δ*cobB* cells (Fig. 1d).

### Δ*cobB* and Δ*ackA*-*pta* mutations affect the size, fluorescence, and processing of IBs

**Fig. 2:**
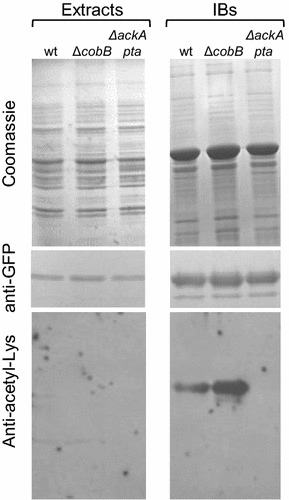
IBs from wt and Δ*cobB* cells contain acetylated VP1GFP. IBs were isolated after 24 h of IPTG induction from the bacteria cultivated, as described in the legend to Fig. 1. The whole-cell extracts and purified IBs were submitted to SDS-PAGE and immunodetection using anti-GFP (Sigma) and anti-acetyl lysine antibodies (Abcam). IBs isolated from equal amounts of cells were loaded onto the gel

**Fig. 3:**
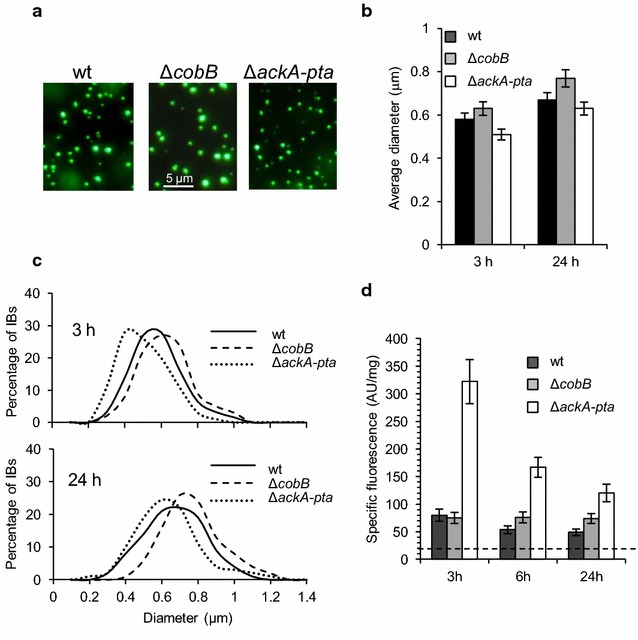
The size of IBs is affected by Δ*cobB* and Δ*ackA*-*pta* mutations. **a** Fluorescence microscopy images of IBs purified from wt, Δ*cobB*, and Δ*ackA*-*pta* cells after 24 h of IPTG-induction. **b, c** Average diameters of IBs isolated after 3 or 24 h of induction were estimated using Zen imaging software (Zeiss). **d** Specific fluorescence emission of VP1GFP in IBs. The *dotted line* indicates specific fluorescence of soluble VP1GFP. *Error bars* represent the standard deviation of three independent experiments

To further characterize the IBs, we immunodetected acetylated lysines in whole cell extracts and in the aggregates (Fig. 2). To allow efficient IPTG induction, the bacteria were grown in Lysogeny broth (LB) without glucose; therefore, acetylated proteins were hardly detectable in whole cell extracts (Fig. 2). Nevertheless, we observed strong acetylation of aggregated VP1GFP in wt cells and significantly stronger acetylation of aggregated VP1GFP isolated from Δ*cobB* cells. According to our expectations, IBs in the Δ*ackA*-*pta* strain were not acetylated. Since VP1GFP retains its fluorescence in IBs [22], it was possible to examine the size of aggregates by fluorescence microscopy (Fig. 3a). We found that the average diameters of IBs isolated from Δ*cobB* and Δ*ackA*-*pta* cells were slightly higher and lower, respectively (Fig. 3b). The differences between strains were more noticeable when the distribution of IBs’ diameters was compared (Fig. 3c). The IBs showed wide ranges of diameters from 0.2 to 1.3 μm. The population of IBs from the CobB-deficient strain was enriched in larger particles (>0.8 μm), whereas smaller IBs (<0.4 μm) were found mainly in Δ*ackA*-*pta* cells (Fig. 3c). In all tested strains, IBs isolated after 24 h were larger than the aggregates formed after 3 h of induction. Next, we found that the specific fluorescence of VP1GFP was significantly higher in IBs than in soluble fractions (Fig. 3d). This difference was particularly striking for the Δ*ackA*-*pta* strain. After 3 h of IPTG induction, we detected ~320 fluorescence AU/mg protein in IBs versus ~20 fluorescence AU/mg protein in the soluble fraction. Specific fluorescence emission of IBs from Δ*ackA*-*pta* cells was fourfold higher than that observed in wt IBs. This might be due to the fact that the IBs from Δ*ackA*-*pta* cells were produced more slowly, were smaller in size, and probably contained more polypeptides with native structures than IBs from the other strains. In wt and Δ*ackA*-*pta* cells, IBs appeared to become less fluorescent over time, whereas in the Δ*cobB* strain, the specific fluorescence remained relatively constant and at the end of the experiment was higher by 30% than in wt IBs. These results may suggest that acetylation could facilitate the retainment of VP1GFP fluorescence. Although specific fluorescence of IBs decreased in Δ*ackA*-*pta* cells with the highest rate, at the end of the experiment, the specific fluorescence of insoluble VP1GFP in this strain was at least twofold higher than that in IBs from wt cells.

**Fig. 4:**
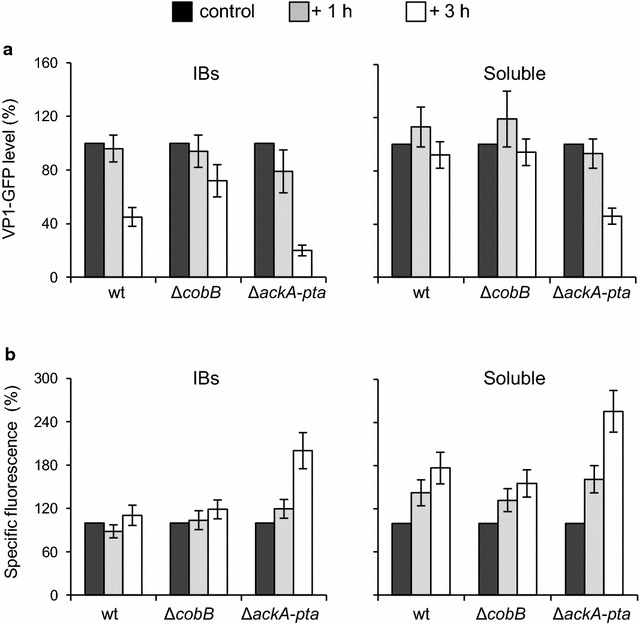
IBs processing is affected by the Δ*cobB* and Δ*ackA*-*pta* mutations. The bacteria were grown and VP1GFP synthesis was induced as described in the legend to Fig. 1. At 3 h after IPTG induction (control), chloramphenicol (200 mg/L) was added to arrest protein synthesis and the cultures were incubated further at 28 °C. **a** After 1 and 3 h (+1 h, +3 h), cell samples were collected to isolate soluble fractions and IBs, which were subsequently analyzed by SDS-PAGE, immunodetection using anti-GFP antibodies, and densitometry. The level of VP1GFP after 3 h of induction (control) corresponds to 100%. **b** GFP fluorescence in soluble fractions and IBs was recorded in EnSpire plate reader (PerkinElmer). Specific fluorescence emission of VP1GFP was estimated as described in the “Methods” section. *Error bars* represent the standard deviation of three independent experiments

In further experiments, we analyzed the processing of IBs after the arrest of protein synthesis by chloramphenicol. We found that the removal of IBs was inhibited in Δ*cobB* cells, but occurred significantly faster in the Δ*ackA*-*pta* strain when compared to wt cells (Fig. 4a). Three hours after the arrest of protein synthesis, 45, 72, and 20% of the initial amounts of IBs remained in wt, Δ*cobB*, and Δ*ackA*-*pta* cells, respectively. In the beginning, an increase in soluble VP1GFP fraction was observed in wt and Δ*cobB* cells, but further incubation resulted in a decrease in the level of soluble VP1GFP due to the degradation of the protein (Fig. 4a). The degradation of soluble VP1GFP was significantly faster in Δ*ackA*-*pta* cells. We observed that during the processing of IBs, the specific fluorescence of VP1GFP increased, particularly in soluble fractions (Fig. 4b). These data suggested that after the arrest of protein synthesis, unfolded but soluble VP1GFP accumulated in the cell was refolded. Again, this effect was drastically enhanced in the Δ*ackA*-*pta* soluble fraction. In addition, a twofold increase in specific fluorescence was detected in IBs isolated from this strain.

The key factors that determine the rate of protein aggregation are the net charge, hydrophobicity and propensity to form a secondary structure [23]. VP1GFP contains 28 lysine residues and at the intracellular pH (pH 7.6) [24] has a net charge of −4. Acetylation of all 28 lysine residues in VP1GFP increases the negative net charge of the protein to −32. This should generate electrostatic repulsions between VP1GFP molecules resulting in increased solubility [25, 26]. However, our results indicated that enhanced VP1GFP acetylation caused faster aggregation of the protein (Fig. 1d). This could be explained by the fact that acetylation increases the hydrophobicity of the lysine side chain which may in turn enhance protein aggregation. It seems that the electrostatic repulsions were not sufficient to completely prevent hydrophobic interactions between VP1GFP molecules. To support our assumption we analysed the tendency of acetylated VP1GFP to form aggregates by submitting modified VP1GFP sequence to Aggrescan software [27]. The algorithm identifies specific fragments in proteins that act as “hot spots” driving aggregation. Lysine residues in the VP1GFP sequence were changed for hydrophobic amino acids: phenylalanine, isoleucine or valine to mimic lysine acetylation. We found that in modified VP1GFP variants new hot-spot areas were created suggesting that acetylated VP1GFP exhibits enhanced tendency to form aggregates (Additional file 1: Figure S1).

### *ΔcobB* and *ΔackA*-*pta* mutations affect the aggregation of endogenous proteins in stationary *E. coli* cells

**Fig. 5:**
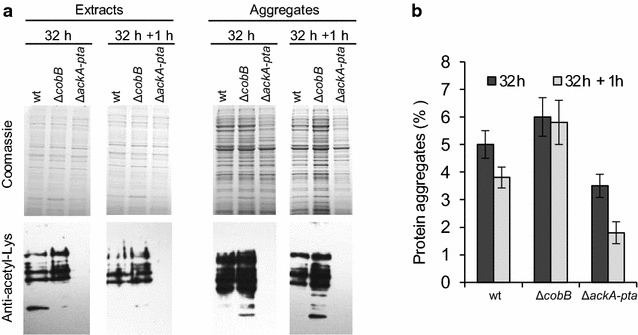
Aggregation of endogenous *E. coli* proteins in Δ*cobB* and Δ*ackA*-*pta* strains. Bacteria were grown at 37 °C in LB medium supplemented with 0.4% glucose. After 32 h, the cells were collected, resuspended in fresh LB medium without glucose, and incubated for 1 h at 37 °C. **a** Protein aggregates were isolated as described in “Methods” section, resolved by SDS-PAGE, and visualized by Coomassie staining or immunodetected using anti-acetyl lysine antibodies (Abcam). Volumes corresponding to equal amounts of bacteria were loaded onto the gels. **b** The level of aggregated proteins in relation to total protein in cell extracts was calculated on the basis of densitometry. *Error bars* represent the standard deviation of three values

Our studies revealed that acetylation or deacetylation can influence the formation and processing of IBs. However, it is not excluded that the observed effect can depend on an overproduced recombinant protein. To extend our studies, we investigated the effect of Δ*cobB* and Δ*ackA*-*pta* mutations on the aggregation of endogenous *E. coli* proteins during the late stationary phase [28]. In this experiment, we used LB supplemented with 0.4% glucose to stimulate protein acetylation [16]. We found that in Δ*cobB* whole cell extract and aggregates, the overall levels of acetylated proteins were increased, whereas in Δ*ackA*-*pta* extracts and aggregates, acetylated proteins were hardly detectable (Fig. 5a). The Δ*cobB* strain produced ~25% more aggregates than wt cells and the Δ*ackA*-*pta* strain contained ~40% less aggregates than wt cells. Under deacetylation-favorable conditions, when the bacteria were transferred to fresh LB medium without glucose, the removal of aggregates was most effective in the Δ*ackA*-*pta* strain (Fig. 5b). These results strongly resembled those obtained for IBs (Figs. 1, 2, 3, 4), suggesting that acetylation or deacetylation of proteins can generally affect the formation and processing of protein aggregates. Surprisingly, partial deacetylation of aggregated proteins was also observed in the Δ*cobB* strain (Fig. 5a). It should be noted that our results were not fully consistent with the data published by Mizrahi et al. which demonstrated that the aggregation of heat denatured proteins was enhanced in *ΔackA*-*pta* cells [13, 14] (see “Discussion” section).

**Fig. 6:**
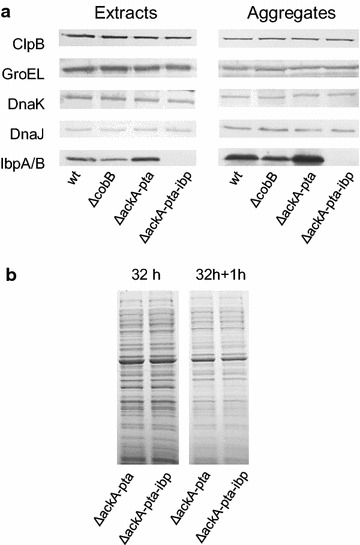
The IbpA/B molecular chaperones are overproduced in Δ*ackA*-*pta* cells. Bacteria were grown as described in the legend to Fig. 5. **a** Whole-cell extracts and purified aggregates were resolved by SDS-PAGE and submitted to immunodetection using antibodies against ClpB, DnaK, GroEL, DnaJ, and IbpA/B molecular chaperones. Both IbpA and IbpB migrated in the polyacrylamide gel as one band. Volumes corresponding to equal amounts of bacteria were loaded onto the gels. **b** Purified aggregates were visualized by Coomassie staining

It was demonstrated that Δ*ackA*-*pta* cells accumulate transcripts of heat shock protein (*hsp*) genes, including *dnaK*, *groEL*, *groES*, and *clpB* [8]. Therefore, we assumed that the faster removal of aggregated proteins in Δ*ackA*-*pta* cells could at least partly result from chaperone activity of overexpressed heat shock proteins (Hsps). Indeed, we found that the Δ*ackA*-*pta* cell extract and aggregates contained increased levels of IbpA/IbpB chaperones when compared to the wt and Δ*cobB* strains (Fig. 6a). Further experiments demonstrated, however, that IbpA/B were not required for the efficient removal of the aggregated proteins. In the stationary phase and under deacetylation conditions, the levels of protein aggregates were comparable in the Δ*ackA*-*pta*-*ibpAB* and Δ*ackA*-*pta* strains (Fig. 6b). We supposed that the lack of IbpA/B in Δ*ackA*-*pta*-*ibpAB* cells could be compensated by the overproduction of DnaK or other molecular chaperones [29–31], but the levels of DnaK, DnaJ, ClpB, and GroEL were similar in all tested strains (Fig. 6a). These results suggest that the removal of aggregates in Δ*ackA*-*pta* cells was enhanced due to the lack of protein acetylation rather than increased activities of chaperones.

## Discussion

In this study, we demonstrated that Δ*ackA*-*pta* and Δ*cobB* mutations exerted different effects on the formation and processing of IBs. We found that IBs isolated from the Δ*ackA*-*pta* strain showed fourfold higher specific VP1GFP fluorescence than their counterparts from wt cells. Previously, Garcia-Fruitos et al. [32] demonstrated that the specific GFP fluorescence can be increased up to threefold in the absence of selected molecular chaperones or proteases. The increase in fluorescence was often accompanied by enhanced aggregation of GFP. This was probably caused by the stabilization of the recombinant protein, due to impaired degradation, and the activity of the protein quality system which supported proper folding of insoluble GFP [32]. In our studies, it was observed that slower VP1GFP synthesis in Δ*ackA*-*pta* cells probably gives the recombinant protein time to fold properly and resulted in postponed aggregation and higher fluorescence (Fig. 1a, c). The lack of toxic effects of acetate could also improve the solubility of VP1GFP and conformational quality of IBs in Δ*ackA*-*pta* cells. This assumption can be supported by our previous studies, showing that externally added acetate strongly stimulated the aggregation of *E. coli* proteins [19]. Conformation of proteins can also be affected by acetylation, leading to the aggregation of modified polypeptides [20, 21]. Our results suggest that diminished acetylation (in Δ*ackA*-*pta*) or enhanced acetylation of proteins (in Δ*cobB* cells) resulted in decreased or increased aggregation in *E. coli*, respectively. Although IBs from the Δ*cobB* strain were characterized by lower specific fluorescence than IBs from Δ*ackA*-*pta* cells (Fig. 3d), they contained higher percentage of total VP1GFP (Fig. 1d), and they were more stable regarding protein level (Fig. 4a) and specific fluorescence (Fig. 3d). It is known that overproduction of recombinant proteins interferes with many physiological processes and causes induction of stress responses in *E. coli* cells [33]. It seems that Δ*cobB* cells coped with this stress better than wt cells and hence overproduced more VP1GFP. This observation is in agreement with the results by Ma and Wood [34], showing that the Δ*cobB* strain exhibits increased oxidative and heat stress resistance. It is believed that soluble misfolded proteins are more toxic for cells than final insoluble aggregates [35]. Therefore, we suppose that faster formation of IBs also enabled Δ*cobB* cells to withstand the stress induced by recombinant protein production. Our results indicated that in the Δ*cobB* strain potentially harmful soluble species of misfolded VP1GFP were sequestered in less toxic IBs faster than in wt cells (Fig. 1d).

The effects observed in the case of endogenous protein aggregates were similar to the results obtained for IBs. In Δ*ackA*-*pta* cells, the formation of aggregates was diminished and the removal of aggregates was increased, whereas the opposite effect was found in Δ*cobB* cells. Surprisingly, under deacetylation-favorable conditions, the aggregates in Δ*cobB* cells were partly deacetylated (Fig. 5a). Recently, a new deacetylase named YcgG, which targets different substrates than CobB, has been identified [36]. Our results may suggest that the aggregates formed during the stationary phase contain CobB-specific as well as YcgG-specific substrates.

We cannot exclude that protein aggregation can be affected indirectly by other physiological changes caused by the loss of AckA-Pta pathway or CobB deacetylase. AcP has been proposed to act as a global transcription regulator, which phosphorylates a number of response regulators [37]. It has been also reported that CobB-controlled acetylation of isocitrate lyase affects the glyoxylate shunt, whereas acetylation of the transcription factor RcsB prevents DNA binding and activates flagella biosynthesis and motility [38].

In our experiments, IBs containing VP1GFP and endogenous protein aggregates formed in stationary *E. coli* cells were efficiently removed in the AcP-deficient strain (Figs. 4, 6). However, Mizrahi and co-workers demonstrated that AcP was required for the proteolysis of some proteins [13, 14]. This discrepancy can be explained by the fact that different proteins and growth conditions were used in our studies and in the experiments described previously [13, 14]. Mizrahi and co-workers found that AcP was required for the removal of truncated soluble forms of homoserine *trans*–succinylase and alkaline phosphatase, and puromycin-derived peptides [13] but not for the degradation of heat shock–denatured luciferase [14]. The Authors observed also enhanced aggregation of endogenous proteins in *ΔackA*-*pta* cells submitted to heat shock [13]. These studies were performed using exponentially growing cells, in contrast to our experiments in which stationary-phase cells were employed. The level of acetylated proteins in exponential-phase cells is very low but increases significantly upon entry into stationary phase [15]. Therefore, we suppose that in exponentially growing cells the effect of the *ΔackA*-*pta* mutation could result from the lack of AcP-dependent phosphorylation rather than impaired protein acetylation.

## Conclusions

Recent studies have demonstrated that lysine acetylation is one of the prevalent post- translational modifications of bacterial proteins. Physiological functions of acetylation/deacetylation of proteins in bacteria are still being discovered. Our studies revealed that diminished and enhanced lysine acetylation can differentially affect the yield, solubility, and biological activity of recombinant proteins. The formation of endogenous protein aggregates during the late stationary phase in *E. coli* cells was also influenced by acetylation. In general, decreased acetylation postponed the formation of endogenous and recombinant protein aggregates, whereas decreased deacetylation stabilized the aggregates. We also found that non-acetylated IBs exhibited significantly higher biological activity than their acetylated counterparts. These findings can be useful for the optimization of the production of recombinant proteins in *E. coli*.

## Methods

### Strains and growth conditions

BW25113 [F^−^, Δ(*araD*-*araB*)567, *lacZ*4787(del)::*rrnB*-3, LAM^−^, *rph*-*1*, Δ(rhaD-rhaB)568, *hsdR*514], as a wild-type strain, and its derivatives BW25113 Δ*cobB* (Keio collection) and BW25113 Δ*ackA*-*pta* were used in this study. The l Red system [39] was used for obtaining Δ*ackA*-*pta* mutant strain. The presence of the deletion was confirmed by PCR. For the overproduction of VP1GFP, the strains were transformed with pTVP1GFP plasmid [22]. The strains were grown at 37 °C in LB medium with agitation (200 rpm.). The strains transformed with the pTVP1GFP plasmid were grown in LB medium supplemented with 100 mg/L ampicillin. To induce overproduction of VP1GFP, isopropyl β-d-thiogalactoside (IPTG) (1 mM) was added to the exponentially growing cultures. To arrest protein synthesis, cultures were treated with 200 mg/L chloramphenicol. Endogenous protein aggregates were isolated from bacterial cultures grown in LB supplemented with 0.4% glucose.

### Isolation of IBs and endogenous protein aggregates

IBs were isolated with CelLytic B, a bacterial cell lysis extraction reagent (Sigma), according to the supplied protocol. For recording GFP fluorescence and fluorescence microscopy, IBs were resuspended in phosphate-buffered saline (PBS).

To isolate endogenous protein aggregates from stationary *E. coli* cultures, cells were pelleted, converted into spheroplasts, and lysed by sonication as described previously [19]. After removal of unbroken cells by centrifugation (15 min, 2000×*g*), the supernatant was incubated with 2% Triton X-100 for 15 min at room temperature. Subsequently, insoluble aggregates were pelleted after 30 min of centrifugation at 21,000×*g* and the remaining supernatant was collected as soluble fraction. Endogenous protein aggregates and whole cell extracts were resolved by SDS-PAGE and analyzed by densitometry to estimate the amounts of aggregated proteins in relation to the total protein content in whole cell extracts (set to 100%).

### SDS-PAGE and western blotting

SDS-PAGE and Western blotting were performed according to Sambrook et al. [40]. Polyclonal rabbit antisera against ClpB, DnaK, DnaJ, GroEL, IbpA/B [29], GFP (Sigma), anti-rabbit peroxidase conjugate (Sigma), and substrates such as 4-chloro-1-naphtol and H_2_O_2_ (Sigma) were used for immunodetection. Acetylated proteins were detected using anti-acetyl-lysine antibodies (Abcam), anti-rabbit peroxidase conjugate (Sigma), and Clarity Western ECL Substrate (Bio-Rad).

### Fluorescence determination

Fluorescence of VP1GFP was recorded in PerkinElmer EnSpire plate reader (excitation, 450 nm; emission, 510 nm). Appropriately diluted whole-cell extracts and soluble fractions and IBs resuspended in PBS were used for these measurements. To determine the specific fluorescence of VP1GFP, amount of VP1GFP protein in IBs, extracts and soluble fractions were estimated after SDS-PAGE and immunodetection.

Epifluorescence micrographs of IBs were taken by using a Zeiss Axio microscope at a spatial resolution of 0.2 µm (excitation wavelength at 450–490 nm and emission wavelength at 515 nm).

## Additional file

**Additional file 1: Figure S1.** Hot-spot area plot predicted by Aggrescan software [27] for VP1GFP. New aggregation hot-spot areas were created in VP1GFP variants in which lysine residues were replaced by hydrophobic aminoacids (F, I or V) to mimic lysine acetylation.

## Abbreviations

AcP: acetyl phosphate
AU: arbitrary units
IBs: inclusion bodies
Hsps: heat shock proteins
