# Supplementary figures and images for: The effect of protein acetylation on the formation and processing of inclusion bodies and endogenous protein aggregates in Escherichia coli cells
Source: Microb Cell Fact. 2016 Nov 10;15:189. doi: 10.1186/s12934-016-0590-8 (PMC5105262; doi:10.1186/s12934-016-0590-8)

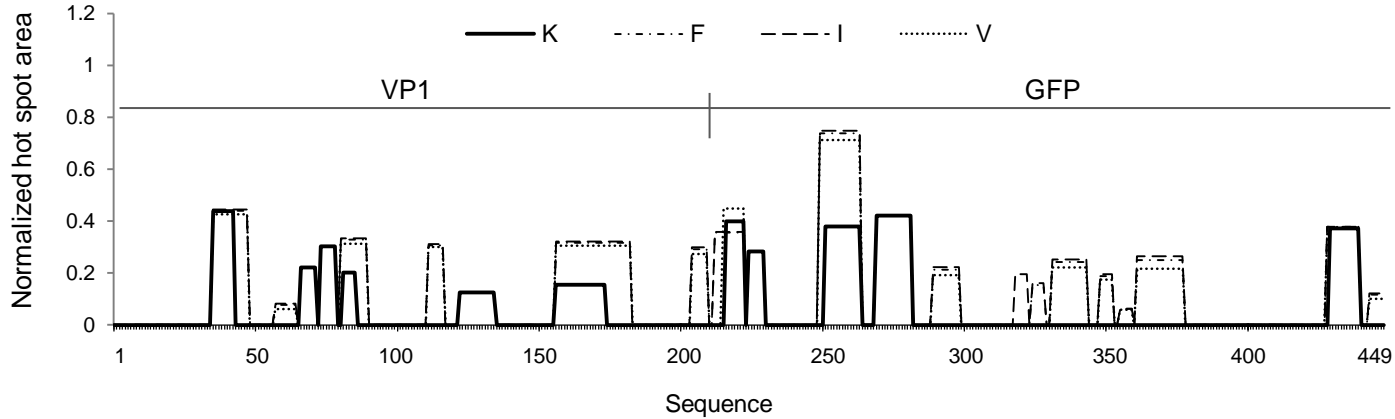

Supplement: Supplementary file 1 — Additional file 1: Figure S1. Hot-spot area plot predicted by Aggrescan software [27] for VP1GFP. New aggregation hot-spot areas were created in VP1GFP variants in which lysine residues were replaced by hydrophobic aminoacids (F, I or V) to mimic lysine acetylation. [file 12934_2016_590_MOESM1_ESM.pdf]
